# Mindfulness-Based Interventions for Psychological Stress in Medical Students: A Systematic Review

**DOI:** 10.3390/healthcare14131961

**Published:** 2026-07-02

**Authors:** Ana Leonor Couto, Daniel Humberto Pozza, Ricardo João Teixeira, Isaura Tavares

**Affiliations:** 1 Faculty of Medicine, University of Porto, 4200-319 Porto, Portugal or analeonorcouto@hotmail.com (A.L.C.); dhpozza@med.up.pt (D.H.P.); 2 Institute for Research and Innovation in Health and IBMC (i3S), University of Porto, 4200-135 Porto, Portugal; 3 CINEICC, Faculty of Psychology and Educational Sciences, University of Coimbra, 3000-115 Coimbra, Portugal; ricardojft@gmail.com; 4 REACH—Mental Health Clinic, 4000-138 Porto, Portugal

**Keywords:** well-being, mental health, healthcare professionals, self-regulation, medical education, stress

## Abstract

**Highlights:**

**What are the main findings?**

- The majority of the reviewed studies (including both randomized and non-randomized trials) suggest that Mindfulness-Based Interventions (MBIs) lowered the psychological stress levels reported by medical students. The studies are heterogenous in what concerns the intervention procedures and experimental design.
- MBIs led to broad improvements in mental health issues, specifically reducing levels of anxiety and depression while improving emotion regulation and overall mindfulness skills.

**What are the implications of the main findings?**

- Medical schools should consider the possibility of increasing the offer of MBIs to their students. This may lead to improvement of psychological resilience of medical students.
- To integrate these tools effectively, researchers must identify the specific dosages and formats of MBIs that offer the greatest benefit within high-intensity academic environments.

**Abstract:**

**Background/Objectives:** Medical students are frequently exposed to high levels of psychological stress due to demanding academic workloads and individual responsibilities. Mindfulness-Based Interventions (MBIs) have been increasingly explored as strategies to improve mental well-being of this population. The objective of this systematic review is to evaluate the effects of MBIs on psychological stress among medical students. **Methods**: A systematic search of three databases (PubMed, Scopus, and Web of Science) was conducted following PRISMA guidelines upon protocol registration in PROSPERO. Randomized controlled trials (RCTs) and non-randomized studies assessing mindfulness interventions in medical students were included. Risk of bias was evaluated using the Cochrane RoB 2 tool for RCTs and the ROBINS-I tool for non-randomized studies. **Results**: Twenty-one studies met the inclusion criteria, including fourteen RCTs and seven non-randomized studies. The types of MBIs were variable between the included studies, including mainly Mindfulness-Based Stress Reduction (MBSR) but also Compassion Cultivation Training (CCT). The interventions were also heterogeneous in what concerns duration and format. **Conclusions**: Although further research is required to address current methodological gaps, namely the variable types, formats, and duration of MBIs, this study suggests putative benefits of integrating mindfulness into medical education to promote psychological resilience.

## 1. Introduction

Medical students are exposed to high levels of stress during training due to the heavy workload and individual responsibilities, along with demanding situations related to patient illness [1,2,3]. This stress is further exacerbated by a competitive, high-pressure academic environment and growing uncertainty regarding future medical careers. Furthermore, poor coping mechanisms during medical school strongly predict inadequate stress management, professional distress, and compassion fatigue later in practice—ultimately compromising both self-care and clinical efficacy [4,5,6,7]. The consequences of poor stress management are multifaceted, ranging from compromised patient care and increased absenteeism to psychological challenges—such as anxiety and depression—that can ultimately culminate in burnout [8,9].

In 1984, Lazarus and Folkman defined psychological stress as a “relationship between the person and the environment that is appraised by the person as taxing or exceeding his or her resources and endangering his or her well-being” [10]. Psychological stress manifests in medical students across multiple domains. Physically, it can induce fatigue, palpitations, and muscle tension, while cognitively, it often impairs concentration and causes forgetfulness or persistent worry. Emotionally, students may experience irritability, depressive symptoms, and social isolation; behaviorally, stress frequently disrupts sleep patterns, alters appetite, and increases the risk of substance use [11]. Academic stress can be defined as a form of psychological stress experienced by students in response to the demands of the academic environment, including heavy workload, time pressure, performance expectations, and adaptation to higher education, which are appraised as challenging or exceeding their coping resources [12]. Psychological stress among medical students is a well-recognized issue, stemming from a distressing emotional and physiological state that occurs when the rigorous demands of medical training exceed their perceived ability to cope [8,9].

According to the most operational definitions, mindfulness consists of “paying attention in a particular way: on purpose, in the present moment, and nonjudgmentally” [13]. Mindfulness can be practiced formally (e.g., structured practices such as focusing on breathing, body scan, sounds or open awareness) and informally (e.g., bringing mindfulness awareness to everyday activities) [14]. There are several Mindfulness-Based Interventions (MBIs) that mostly combine formal and informal practices. The most well-known program is the Mindfulness-Based Stress Reduction (MBSR) educational program, created by Jon Kabat-Zinn [15,16]. The MBSR program is a structured, eight-week course that includes both formal and informal practices [15,16]. It aims to shift participants from a state of automatic reactivity (the stress response) to a state of conscious responding. The MBSR program typically consists of weekly group classes, lasting for 2.5/3 h each, along with a mandatory 6–7 h silent retreat [15,16]. Participants are also assigned daily home practices that progressively increase in duration. The intervention incorporates somatic exercises, such as the body scan and mindful stretching, alongside practices designed to cultivate cognitive awareness, specifically through seated meditation. The participant is also trained in foundational attitudes such as “non-judging”, “patience”, and “acceptance” [15,16]. In the wake of MBSR’s development, subsequent programs have evolved to more explicitly integrate compassion, drawing deeply upon this central pillar of Buddhist philosophy. Compassion is a core principle of Mindfulness practices directed to the one who practices Mindfulness meditation, namely Mindfulness Self-Compassion [MSC; [17]], but also to other sentient beings. Another commonly used MBI is Compassion Cultivation Training (CCT), which is also an 8-week program designed to cultivate compassion, kindness, and well-being [18]. Furthermore, multiple shorter mindfulness-based courses have been developed [19], as well as audio-guided and online programs [20,21]. In spite of their heterogeneity, all MBIs share common principles. The mind is invited to rest and focus in an anchor (e.g., breath, body sensations) and to replace its “natural features”, such as being busy and judgmental, by acceptance and open curiosity. This may lead to decentering and emotional regulation [19]. It should, however, be noticed that MBIs are very heterogenous in their structure, which leads to challenges in comparing the results of different studies, as shown by systematic reviews and meta-analyses [22]. Findings from a specific MBI cannot be universally applied to different demographic populations or healthcare.

Since it is important to focus the studies of the effects of MBIs on specific populations and given the profound impact of stress on both emotional and physical health of medical students, there has been growing interest in mindfulness interventions in contexts of medical education. It is important to help medical students cope with mental and emotional challenges during the medical course and also develop a best stress managing in their future as healthcare professionals [23,24,25]. Evidence from systematic reviews suggests that MBIs may improve medical students’ well-being by reducing stress, anxiety, and depression as well as improving mindfulness skills [23,26,27]. However, these studies were published several years ago and may not reflect the most recent evidence. Furthermore, findings across reviews remain heterogeneous, with variability in type of intervention, participants’ characteristics, and outcome measures. It is important to focus on the specificities of the medical student population. This variability highlights the need for a more comprehensive and rigorous synthesis of the existing literature to better understand the potential benefits and limitations of MBIs in reducing psychological stress in medical students.

Herein, the main objective of this systematic review is to evaluate and synthesize the current evidence on the effects of MBIs on medical students’ psychological stress. By focusing exclusively on this population, this review seeks to identify the main practices and findings of the current literature and determine if it is possible to support the development of mindfulness strategies to promote improved mental health outcomes of medical students.

## 2. Materials and Methods

This systematic review adhered to the Preferred Reporting Items for Systematic Reviews and Meta-Analyses (PRISMA) guidelines. To ensure transparency and reproducibility, the search followed a protocol registered in the PROSPERO database (ID: CRD420251184089). The protocol is publicly available for consultation, and it was reviewed and approved by all the authors. The review’s PICO question was: “In medical students (P), do mindfulness interventions (I) as compared with usual care or other interventions (C) reduce psychological stress (O)?”.

A search in three electronic bibliographic databases, namely PubMed, Scopus, and Web of Science, was carried out on 15th December 2025. The search strategy was built up combining search words (Mindfulness, Medical Students, Psychological Stress) and MeSH terms (Mindfulness; Students, Medical; Stress, Psychological). The search strategy used for PubMed was the following: (“Mindfulness”[Mesh]) AND (“Students, Medical”[Mesh]) AND (“Stress, Psychological”[Mesh]); for Scopus, we used: “Mindfulness” AND “Students, Medical” AND “Stress, Psychological”; and for Web of Science, we used: Mindfulness AND Medical Students AND “Psychological Stress” (All Fields).

To be included in the following steps of the analysis, the studies should follow the inclusion criteria: (1) medical students who have completed a mindfulness intervention; (2) measures of psychological stress levels and/or psychological stress-related outcomes; (3) publishing in English; and (4) Randomized Controlled Trials (RCTs) or non-randomized studies.

The exclusion criteria were as follows: (1) studies involving non-medical students or mixed populations in which the specific effects on medical students were not reported separately from the other student populations; (2) studies with medical students that had not completed a mindfulness intervention; (3) studies that did not include measures of psychological stress levels and/or psychological stress-related outcomes; and (4) duplicated studies, systematic reviews, opinion papers, commentaries, and studies focusing on the effects of the COVID-19 pandemic. These latter studies were excluded to minimize methodological variability within the current systematic review. Conceptually, research conducted during the COVID-19 pandemic spanned vastly different phases—such as lockdown versus non-lockdown periods—and frequently carried limitations, including small sample sizes, a lack of active controls, and a reliance on online interventions. The inherent challenges of generalizing and extrapolating data from such a unique historical period further justified this exclusion criterion.

Titles and abstracts of the identified records were independently screened by two reviewers using the Rayyan tool [28] to determine eligibility. To prevent reviewers from being influenced by each other’s decisions and reduce bias, the “blind mode” on the Rayyan platform was used during all of the screening process. Subsequently, full texts of potentially relevant studies were reviewed in detail. The degree of concordance between the reviewers was evaluated using Cohen’s Kappa statistic [29]. Data were extracted from the eligible studies in a collaborative manner. The information extracted included authors, year, study design, country, sample size, participants’ characteristics (age, gender, and year of training), type of mindfulness intervention, control, duration of intervention, main results (psychological stress based on the query defined above), secondary outcomes (mostly related to anxiety, depression, and other mental health issues), along with the main limitations of the studies. The main focus was psychological stress and not mental issues, although reduction of stress is important to reduce anxiety and depression.

The primary outcomes were based in psychological stress, which is frequently associated with mental health issues, such as depression and anxiety. The latter were mostly analyzed as secondary outcomes in the current systematic review.

The risk of bias of randomized controlled trials was assessed with the Cochrane RoB 2 tool [30], whereas non-randomized studies were evaluated with the ROBINS-I tool [31]. Results were visualized using the Risk-of-bias VISualization (robvis) tool [32].

Finally, a narrative synthesis was conducted to summarize the findings of the included studies.

## 3. Results

**Figure 1:**
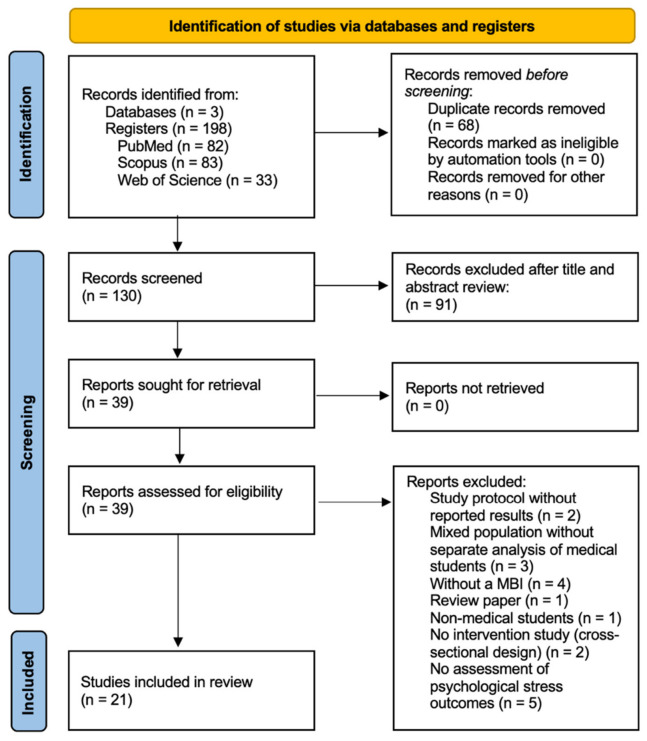
PRISMA 2020-compliant flow diagram of the study selection process, including identification, screening, eligibility, and inclusion of studies.

The PRISMA flow diagram outlining the selection of included studies is shown in Figure 1.

The literature search resulted in 198 records identified from three databases (82 from PubMed, 83 from Scopus and 33 from Web of Science). After removing 68 duplicate records, 130 articles remained for screening. Of these, 91 were excluded after title and abstract review for not fulfilling the inclusion criteria and/or meeting the exclusion criteria.

A total of 39 reports were selected for full-text examination. Of these, 18 were excluded for the following reasons: 2 were study protocols without reported results; 3 had a mixed population without separate analysis of medical students; 4 did not have a Mindfulness-Based Intervention (MBI); 1 was a review paper; 1 included non-medical students; 2 did not include an intervention; and 5 did not assess psychological stress outcomes.

Ultimately, 21 studies were included in this review, comprising 14 randomized controlled trials (67%) and 7 non-randomized studies (33%).

The Kappa coefficient was 1.00, indicating perfect agreement between the reviewers.

### 3.1. Description of Included Studies

The core characteristics of the 21 studies included in this systematic review are presented in detail in Table 1. A total of 2484 students were included in the data analysis of the selected studies. All of the studies that specified the year of the medical course included undergraduate students. The studies mostly assessed the effects of MBSR (*n* = 8), CCT (*n* = 3), and other MBIs (*n* = 10). The results are presented based on the type of mindfulness intervention. The most common used questionnaire to evaluate psychological distress was the Perceived Stress Scale (PSS), which was used in 15 studies (71%), followed by the Depression, Anxiety, and Stress Scale-21 (DASS-21, which was used in 3 studies (14%). The secondary outcomes were much more variable and mostly related to mental health issues. The results of the studies are summarized based on the intervention type, duration, and main limitations.

#### 3.1.1. Intervention Type

MBSR programs represent the most frequent evaluated intervention, with eight articles focusing on its effects, followed by three studies using cultivation compassion training (CTT). Regarding MBSR, most interventions followed the standard format of 8 weeks, with some variations such as a 10-week program or six 2-h courses [33,34]. Most trials compared MBSR with a waitlist or an active control group. Two studies did not include a control/comparison group [34,35].

As the primary focus of this analysis, MBSR demonstrated an impact on stress reduction. Significant reductions in the PSS from baseline to post-intervention was reported [35,36]. However, in the study conducted by Yay-Pence et al. (2025), there were no significant differences between the intervention and control [36]. Lampe et al. (2021) demonstrated a protective effect, as the MBSR group maintained stable stress levels, while the control group showed a significant increase [34]. In contrast, Moore et al. (2024) and Alzahrani et al. (2023) found no significant differences between intervention and control groups in stress scores [37,38]. Additionally, in the study conducted by van Dijk et al. (2017), the MBSR group reported lower levels of psychological distress during the follow-up period, as evaluated using the Brief Symptom Inventory (BSI) [39]. Summarizing the present findings in what concerns the main outcome (stress reduction), all the compassion-based interventions [18,40,41] decreased stress, which also occurred with the majority of MBSR interventions [32,34,37,38,39]. The large majority of interventions lasted for 8 weeks [18,32,33,34,35,36,37,38,39,42,43], but the efficacy in stress reduction was variable. As to the effects of brief/online interventions [43,44,45] the studies were scarce, and it is not possible to draw any conclusions.

MBSR influenced other mental health dimensions. For example, Yan et al. (2024) reported significant reductions in anxiety (Generalized Anxiety Disorder-7—GAD-7), depression (Patient Health Questionnaire-9—PHQ-9), and posttraumatic stress symptoms (Posttraumatic Stress Disorder Checklist from the DSM-5—PCL-5), post MBSR [40]. In the study conducted by Yay-Pence et al. (2025), both intervention and control groups showed significant reductions in depression and anxiety (HADS) but MBSR was significantly more effective than Cognitive Behavioral Stress Reduction (CBSR) in reducing anxiety in per-protocol analysis [36]. Furthermore, Rosenzweig et al. (2003) reported a significant reduction in tension-anxiety, as measured by the Profile of Mood States (POMS) [33]. However, Alzahrani et al. (2023) showed no significant differences between groups in the PHQ-9 and GAD-7 [38].

Regarding mindfulness skills, a significant increase was observed across several scales, including the Mindful Attention and Awareness Scale (MAAS) (Yan et al., 2024; Lampe et al., 2021), the Freiburg Mindfulness Inventory (FMI) (Moore et al., 2024), and the Five Facet Mindfulness Questionnaire (FFMQ) (van Dijk et al., 2017) [34,37,39,40]. However, Alzahrani et al. (2023) reported that there were no significant differences between groups in the MAAS [38].

In the study conducted by van Dijk et al. (2017), medical students reported improvements in mental health (Mental Health Continuum-Short Form—MHC—SF) and life satisfaction (Life-Satisfaction Questionnaire-9—LiSat-9) and less dysfunctional cognition (Irrational Beliefs Inventory—IBI), which were sustained over a 20-month follow-up period [39]. Yan et al. (2024) also showed improvements in resilience, evaluated with the Connor-Davidson Resilience Scale-10 (CD-RISC-10), and in life satisfaction, assessed with the Satisfaction with Life Scale (SWLS) [40].

Finally, Erogul et al. (2014) revealed a significant improvement in self-compassion, assessed with the Self-Compassion Scale (SCS), although participants did not achieve higher scores on the Resilience Scale (RS) when compared to the control group [35].

Of the three studies that assessed the effects of CCT, two (Rodriguez-Moreno et al., 2024; Rojas et al., 2023) were derived from the same clinical trial conducted with 40 medical students in Spain [41,46]. Overall, CCT showed a significant effect on reducing psychological stress, as evaluated using the Depression, Anxiety, and Stress Scale-21 (DASS-21). Weingartner et al. (2019) substantiated these findings through qualitative reports of stress reduction, despite methodological limitations, such as a small sample size and the absence of a control group [18].

Beyond the impact on stress, CCT showed significant improvements in mindfulness skills as evaluated by the Five Facet Mindfulness Questionnaire (FFMQ) and the Kentucky Inventory of Mindfulness Skills (KIMS). Additionally, Rodriguez-Moreno et al. (2024) highlighted that the development of these mindfulness facets acted as a mediator for anxiety reduction [41].

Furthermore, the Spanish trial conducted by Rodriguez-Moreno et al. (2024) reported significant improvements in emotion regulation, assessed with the Difficulties in Emotion Regulation Scale—DERS. Rojas et al. (2023) showed a notable decrease in burnout (evaluated with the Maslach Burnout Inventory-Student Survey—MBI-SS) as well as greater self-compassion (evaluated with the Self-Compassion Scale-Short Form—SCS-SF) [46].

The CCT interventions varied from five to eight weeks. In the randomized trials, the intervention groups were compared to a waitlist control group.

In this review, ten studies used MBIs that were more general, shorter, or did not define a structured protocol. Fazia et al. (2023) used 10 twice-a-week Integral Meditation classes (e.g., breath, posture, etc.), combined with dietary recommendations and yoga sessions [47]. Kakoschke et al. (2021) implemented a 5-week program combining formal mindfulness meditation with informal practices that integrate mindfulness into daily life [44].

Both interventions were effective in reducing perceived stress (PSS) [44,47]. Moreover, Fazia et al. (2023) described improvements in resilience (using the Resilience Scale-14—RS-14), mental well-being (with the Warwick-Edinburgh Mental Wellbeing Scale—WEMWBS), and emotion regulation (applying the DERS) as well as a reduction in mind wandering (with the Mind Wandering Spontaneous—MW-S) and in overall distress (Positive and Negative Affect Schedule—PANAS) [47]. Similarly, Kakosche et al. (2021) reported significant increases in mental health (MHC-SF), mindfulness (FMI), and study engagement (Utrecht Work Engagement Scale for Students—UWES-S) from pre- to post-intervention [44].

Ray et al. (2025) [48] conducted a 12-week online mind-body wellness program with weekly sessions on meditation (e.g., grounding and letting go) combined with breathwork, yoga, and nutrition. A large majority of medical students (74.8%) found this program accessible and satisfying (scale 0–100). Despite a high dropout rate, the program led to reductions in stress (PSS-10), anxiety (HADS), and depression (PHQ-9), alongside improvements in mindfulness (FFMQ) and quality of life (1–100 Self-Assessment Scale. Waetcher et al. (2021) reported significant improvements in PSS and anxiety, as measured by the State-Trait Anxiety Inventory (STAI), through a 12-week wellness intervention involving mindfulness, yoga, or walking [49]. Regarding mindfulness, the roots in the three-step process outlined by Tibetan teacher Yongey Mingyur were followed (relaxation, stabilization of attention, and training for clarity). In contrast, Neto et al. (2020) found no significant differences in stress, anxiety, and depression (DASS-21), quality of life (World Health Organization Quality of Life—WHOQOL-BREF), or mindfulness (FFMQ) after a 6-week mindfulness meditation course, but the authors outlined limiting data on adherence as a confounding factor [42].

Regarding interventions delivered via audio, Warnecke et al. (2011) conducted an 8-week intervention, using guided practice CDs, that reported reduced stress (PSS) and anxiety (DASS), with effects maintained at follow-up [45]. Yang et al. (2018) [50] observed that a 30-day audio-guided program, with short sessions (10–20 min per audio), significantly decreased perceived stress (PSS) and increased general well-being (General Well-Being Scale—GWBS). The program included guidance about awareness of body at rest, breathing with intention, and breathing normally to noticing sounds, sensing emotions, and acknowledging thoughts without assigning judgment. No consistent significant between-group differences were observed in mindfulness levels (FFMQ).

Other protocols produced diverse outcomes. Moore et al. (2020) [51] showed a significant reduction in stress (PSS) and an increase in self-compassion (SCS) at a 4-month follow-up after an 8-week Mindfulness Training Program (MTP). By the end of the program, 50% of participants practiced mindfulness at least weekly, with 32% maintaining this frequency four months later. Phang et al. (2015) found that the Mindfulness-Based Stress Management program led to reductions in stress (PSS) and mental distress (General Health Questionnaire—GHQ), as well as improvements in mindfulness (MAAS) and self-efficacy (General Self-Efficacy—GSE), though only self-efficacy remained significant after six months [43]. Contrarily, the SMART (Stress Management and Resilience Training) program evaluated by Dyrbye et al. (2017) showed an increase in perceived stress (PSS) and decreases in mental quality of life (Medical Outcomes Study Short Form-8—SF-8) and happiness [52].

#### 3.1.2. Duration

Since the duration of the intervention may be an important aspect to evaluate the efficacy of an intervention, we highlight some of the findings described above based on the duration. The classic 8-week duration intervention was the most common (43%), mainly those based in structured programs, such as MBSR [32,33,34,35,36,37,38,39]. Some studies used variable durations of interventions, namely 5–8 weeks or 10–12 h [18,50]. This also represents a trend to decrease the duration of the interventions, and only 3 out of the 21 studies expanded the duration over 8 weeks, using interventions of 10 or 12 weeks [44]. Very short interventions were used by some studies, namely about 6 h [46]. Based on the results referred above (Section 3.1.1) and presented in Table 1, it is not possible to establish a causal relation between the duration of the intervention and the effects of the MBIs on psychological stress.

#### 3.1.3. Limitations of the Studies

Table 1 also summarizes the main limitations of the studies. Small sample sizes, moderate adherence to the intervention, short follow-up period, lack of blindness, and absence of active control groups are the main constrains of the studies. Another limitation may derive from a possible conflict of interests since in some studies the instructor of the sessions was also the principal investigator [33,42,51].

**Table 1:** Overview of the characteristics of the included studies, ordered by type of most common mindfulness intervention (MBSR, CCT and other MBIs).

Reference	Study Design	Country	Sample Size	Participants’ Characteristics			Type of Mindfulness Intervention	Control	Duration of Intervention	Main Results	Secondary Outcomes	Limitations
				Age (Years)	Gender	Year of Training				(Psychological Stress)		
Yay-Pence, A. et al. (2025) [32]	RCT	Turkey	253	Mean age: 21.85	77% female	N/A	MBSR	CBSR	8 weeks	MBSR and CBSR showed similar reductions in perceived stress scores (PSS).	Significant reductions in depression and anxiety in both groups; MBSR more effective than CBSR in reducing anxiety (HADS).	High attrition rate; lack of long-term follow-up; minimal selection bias; mostly female sample; absence of home practice monitoring.
Moore, S. et al. (2024) [33]	RCT	Australia	101	Mean age: 25 (intervention)/25.5 (control)	Intervention: 26% male, 74% female; Control: 25% male, 75% female.	1 to 4	MBSR	WL	8 weeks	No significant differences in PSS.	Significant improvements in mindfulness (FMI) at post-intervention. No between-group differences at 6 months.	Low response rate; self-reported measures; facilitator was the principal investigator; exclusions due to technical error; lack of control over engagement in other formal mindfulness activities.
Yan, X. et al. (2024) [34]	RCT	China	88	Mean age: 19.98 (intervention)/19.61 (control)	Intervention: 63.46% male; Control: 55.56% male.	1 to 4	MBSR	Eight psychological health education courses and physical training	8 weeks	MBSR significantly reduced psychological stress-related symptoms, particularly anxiety (GAD-7), depression (PHQ-9), and posttraumatic stress disorder symptoms (PCL-5) with maintenance of effects at 1-month follow-up compared to the control group.	Significant improvements in resilience (CD-RISC-10), posttraumatic growth (PTG), life satisfaction (SWLS), and mindfulness (MAAS) compared to the control group.	Participants recruited from a single university limited the number of assessment timepoints.
Alzahrani, A.M. et al. (2023) [35]	RCT	Saudi Arabia	71	Mean age: 22.15 (intervention)/22.44 (control)	Intervention: 38.5% male, 61.5% female; Control: 33.3% male, 66.7% female.	1 to 5	MBSR	WL	8 weeks	No significant differences between the study groups in PSS. At the 3-month follow-up, the MBSR group showed a slightly lower PSS score.	No significant differences between the study groups in the PHQ-9, MAAS and GAD-7. At follow-up, the MBSR group had a significantly lower mean GAD-7 score.	Difficulties to convince medical students; the recruitment phase was interrupted by the COVID-19 pandemic.
Lampe, L.C. et al. (2021) [36]	Non-randomized controlled trial	Germany	136	Mean age: 20.4	Intervention: 90.2% female; Control: 75.8% female	1 to 2	MBSR	No intervention	Six 2-h courses	PSS-10 scores increased significantly in the control group from T1 (baseline) to T3 (6 months later). In the MBSR group, PSS-10 scores showed a non-significant increase.	MAAS decreased significantly in the control group compared to the MBSR group, in which no significant change was observed.	Non-randomized design; small sample size of the intervention cohort; both the intervention and observation period were relatively short.
van Dijk, I. et al. (2017) [37]	RCT	Netherlands	167	Mean age: 23.7 (intervention)/23.3 (control)	Intervention: 72% female; Control: 85% female.	4	MBSR	CAU	8 weeks	Lower levels of psychological distress (BSI) in the MBSR group during the 20-month follow-up period.	Higher levels of positive mental health (MHC-SF) and life satisfaction (LiSat-9), more mindfulness skills (FFMQ), and less dysfunctional cognitions (IBI) compared with CAU during the 20-month follow-up. The MBSR and CAU groups did not differ in physician empathy (JSPE).	Single medical school in Netherlands, which might limit generalizability; lack of control group; potential cross-contamination between groups; lack of booster sessions during follow-up; potential influence of holiday timing on 15-month follow-up assessments.
Erogul, M. et al. (2014) [38]	RCT	USA	58	Mean age: 23.6 (intervention)/23.3 (control)	Intervention: 42.9% female; Control: 48.3% female.	1	MBSR	No intervention	8 weeks	The MBSR group showed significant reduction on PSS, but not at 6 months post-study.	Significant increase on SCS both at the end of the study and at 6 months post-intervention. Participants in the treatment group did not achieve significantly higher scores on RS either at the conclusion of the study or at 6 months post-intervention.	Randomization before consent may have introduced selection bias and limited generalizability; lack of active control; self-reported measures.
Rosenzweig, S. et al. (2003) [39]	Non-randomized cohort-controlled	USA	302	N/A	N/A	2	MBSR	Complementary and alternative medicine seminar	10 weeks	The MBSR group showed significant reductions in tension–anxiety, confusion–bewilderment, and total mood disturbance (TMD). The control group showed increased tension-anxiety, fatigue-inertia, and TMD.	N/A	Lack of randomization; none of the groups may be truly representative of the general medical student population; only a single instrument (POMS) was used as an outcome measure.
Weingartner, L.A. et al. (2019) [18]	Pilot feasibility, mixed methods (pre-post)	USA	45	N/A	N/A	2 and 4	CCT	N/A	5 to 8 weeks	Qualitative reports of reduced stress.	Improvements in mindfulness (KIMS: observing and accepting without judgment).	Small sample; short-term analysis; low response rate; no comparison/control group; data collection varied across cohorts; self-reported outcomes; pre-clerkship students had limited patient interactions; participants self-selected.
Rodriguez-Moreno, S. et al. (2024) [40]	RCT	Spain	40	Mean age: 23.4	92.5% female	N/A	CCT	WL	8 weeks	CCT (compared to the WL) led to significant reduction in psychological stress (DASS-21) at post-intervention. Effects were not maintained at 2-month follow up.	CCT (compared to the WL) led to significant reduction in anxiety (DASS-21) mediated by improvements in emotion regulation (DERS) and in mindfulness skills (FFMQ). Emotional confusion (DERS) was the only mediator that maintained its influence on anxiety symptoms at follow-up.	Moderate sample size was; predominantly female; self-report measures; participants aren’t blinded to the intervention; lack of sustained significant changes and mediators at follow-up.
Rojas, B. et al. (2023) [41]	Pilot RCT	Spain	40	Mean age: 23.4	92.5% female	1 to 6	CCT	WL	8 weeks	CCT (compared to the WL) showed a significant decrease in psychological distress (DASS-21).	CCT (compared to WL), had significant improvements in self-compassion (SCS-SF), mindfulness (FFMQ), and emotion regulation (DERS) as well as significant decreases in burnout (MBI-SS). No significant changes in compassion to others (CSP), empathy (IRI), general well-being (PHI), and resilience (BRS).	Sample size was moderate; use of a waitlist control group instead of an active control comparison; reliance on self-reported measures; impossibility of blinding participants due to the nature of the CCT intervention.
Fazia, T. et al. (2023) [46]	RCT	Italy	362	N/A	N/A	N/A	MBIs	No intervention	10 classes, 35 min each	MBI was effective in reducing perceived stress (PSS) compared to the control.	MBI showed improvements in resilience (RS-14), mental well-being (WEMWBS), emotion regulation (DERS), and attentional control (AC-S and AC-D). Reduction in mind wandering (MW-S) and overall distress (PANAS) compared to the control group.	Voluntary participation; self-reported measures.
Kakoschke, N. et al. (2021) [47]	Observational (pre-post)	Australia	205	Mean age: 18.60	60% female (of the 310 initially enrolled)	1	MBIs	N/A	5 weeks	Reduction in PSS from pre- to post-intervention.	Increase in mental health (MHC-SF), mindfulness (FMI), and study engagement (UWES-S) from pre- to post-intervention.	Multiple lifestyle factors; Lack of a control group; Compulsory program; pre-pot study design; short-term follow up.
Ray, C. et al. (2025) [44]	Pilot feasibility, mixed methods (pre-post)	Canada	64	22 to 28	73% female (of the 74 initially enrolled)	1 to 4	Online mind-body wellness program (yoga, breathwork, meditation, nutrition)	N/A	12 weeks	Reduction in PSS-10.	Reduction in anxiety (14% in HADS) and depression (20% in PHQ-9). Improvements in mindfulness (5.6% in FFMQ) and in the quality of life (5.4% in 1–100 Self-Assessment Scale).	Conducted during COVID-19; high dropout rate; mostly female sample; not powered to evaluate efficacy or long-term program benefits.
Neto, A.D. et al. (2020) [48]	RCT	Brazil	141	Mean age: 18.87 (intervention)/19.07 (control)	Intervention: 48.6% male, 51.4% female; Control: 50.7% male, 49.3% female.	1	Mindfulness Meditation Course	Course about organizational aspects of medical school	6 weeks	No significant differences between intervention and control groups in stress scores (DASS-21).	Anxiety and depression (DASS-21), quality of life (WHOQOL-BREF), and mindfulness (FFMQ) were all non-significant.	Limited data on adherence to mindfulness practice; single-center study; intervention conducted early in the semester; short follow-up period; potential contamination between groups.
Waechter, R. et al. (2021) [49]	RCT	USA	70	Mean age: 25.7	41% male (of the 101 initially enrolled)	1	Wellness intervention program (yoga, mindfulness, or walking)	No intervention	12 weeks	Significant improvement in state anxiety (STAI) and PSS in comparison to control.	Wellness intervention might also influence psychological distress (GHQ-12) and depression (CES-D), as seen by the moderate effect size in scores between the wellness and control groups	Small sample size; population may not be representative of the medical students; attrition from pre- to post-assessment, particularly in the intervention group; self-selection bias (students who dropped out tended to be more anxious).
Moore, S. et al. (2020) [42]	Pilot feasibility, mixed methods (pre-post)	Australia	47	Mean age: 26.7	19.1% male; 80.9% female.	5	Mindfulness Training Program (MTP)	N/A	8 weeks	Significant reduction in participants’ perceived stress levels (PSS) at 4-month follow up.	Significant increase in self-compassion (SCS) at both 8 weeks and 4-month follow-up.	Pilot study without a control group; self-selected sample; small sample size; low response and completion rates; Participants had relatively low PSS and high SCS scores at baseline; program facilitator was the principal investigator; informal mindfulness practice only assessed qualitatively.
Yang, E. et al. (2018) [45]	RCT	USA	88	Mean age: 25.11	36.4% male; 63.6% female	1 to 4	Audio-guided mindfulness meditation program	No intervention	30 days	Significant decrease in PSS in the intervention group from T1 (baseline) to T3 (60 days) compared to the control group.	General well-being (GWBS) significantly increased in the intervention group compared to the control group from baseline to 30 days, increase sustained during 60 days.	Selection bias and response bias due to self-report questionnaires;
Dyrbye, L.S. et al. (2017) [50]	Quasi-experimental	USA	44 (cohort 1) and 22 (cohort 2)	Age: 77.1% <25 years (cohort 1) and 79.5% <25 years (cohort 2)	58.3% female (cohort 1) and 59.1% female (cohort 2)	1	Stress Management and Resilience Training (SMART)	N/A	10–12 h of sessions delivered throughout the first academic year	Increase in PSS.	Significant decreases in mental quality of life (SF-8) and happiness, with no significant changes in burnout (MBI) or resilience (CD-RISC).	Small sample; reduced response rate; the SMART curriculum was added onto existing curricular demands; low frequency of sessions (monthly sessions); students may not be representative of the general medical student population
Phang, C.K. et al. (2015) [51]	RCT	Malaysia	75	Mean age: 21.14 (intervention)/20.94 (control)	Intervention: 30% male, 70% female; Control: 18% male, 82% female.	1 to 3	Mindfulness-Based Stress Management (MBSM)	No intervention	5 weeks	Significant reductions in PSS at 1-week post-intervention. These reductions were not maintained six months after the intervention.	Compared to the control group, the intervention group reported significant reductions in mental distress scores (GHQ) and improvements in mindfulness (MAAS) and self-efficacy (GSE) at 1-week post- intervention. Six months after intervention, only the increase in self-efficacy scores remained significant.	Small sample size; the effectiveness of the intervention was overall not sustained after 6 months (except for self-efficacy measure); randomization only among volunteers; lack of active control group; the investigator (first author) of the study was also the trainer in the intervention.
Warnecke, E. et al. (2011) [43]	RCT	Australia	65	Mean age: 23.4 (intervention)/24.4 (control)	Intervention: 74.2% female; Control: 55.9% female.	Medical students in their final 2 years of their degree course	Audio compact disc (CD) of guided mindfulness practice	No intervention	8 weeks	Significant reduction in the PSS compared to the control group. The effect was maintained 8 weeks after the intervention.	The intervention group showed a significant reduction in the anxiety component of the DASS. Follow-up at 8 weeks post-trial revealed that the effect was maintained.	Small sample size; moderate adherence to the intervention; short follow-up period; absence of physiological measures of stress; inability to blind participants due to the nature of the intervention.

Legend: AC-D, Attention Control Distraction; AC-S, Attention Control Shifting; BRS, Brief Resilience Scale; BSI, Brief Symptom Inventory; CAU, Clinical Clerkships as Usual; CBSR, Cognitive Behavioural Stress Reduction; CCT, Compassion Cultivation Training; CES-D, Centre for Epidemiologic Studies Depression Scale; CSP, Compassion Scale Pommier; CD-RISC-10, Connor-Davidson Resilience Scale-10; DASS-21, Depression, Anxiety and Stress Scale-21; DERS, Difficulties in Emotion Regulation Scale; FFMQ, Five Facet Mindfulness Questionnaire; FMI, Freiburg Mindfulness Inventory; GAD-7, Generalized Anxiety Disorder-7; GHQ-12, General Health Questionnaire-12; GSE, General Self-Efficacy; GWBS, General Well-Being Scale; HADS, Hospital Anxiety and Depression Scale; IBI, Irrational Beliefs Inventory; IRI, Interpersonal Reactivity Index; JSPE, Jefferson Scale of Physician Empathy; KIMS, Kentucky Inventory of Mindfulness Skills; LiSat-9, Life-Satisfaction Questionnaire-9; MAAS, Mindful Attention and Awareness Scale; MBIs, Mindfulness-Based Interventions; MBI, Maslach Burnout Inventory; MBI-SS, Maslach Burnout Inventory-Student Survey; MBSR, Mindfulness-Based Stress Reduction; MHC-SF, Mental Health Continuum-Short Form; MW-S, Mind Wandering Spontaneous; PANAS, Positive and Negative Affect Schedule; PCL-5, Posttraumatic Stress Disorder Checklist from the DSM-5; PHI, Pemberton Happiness Index; PHQ-9, Patient Health Questionnaire-9; POMS, Profile of Mood States; PSS-10, Perceived Stress Scale-10; PTG, Posttraumatic Growth Inventory; RCT, Randomized Controlled Trial; RS-14, Resilience Scale-14; SCS, Self-Compassion Scale; SCS-SF, Self-Compassion Scale-Short Form; SF-8, Medical Outcomes Study Short Form-8; STAI, State-Trait Anxiety Inventory; SWLS, Satisfaction with Life Scale; UWES-S, Utrecht Work Engagement Scale for Students; WEMWBS, Warwick-Edinburgh Mental Wellbeing; WHOQOL-BREF, World Health Organization Quality of Life; WL, Waitlist; N/A, Not applicable.

### 3.2. Risk of Bias

**Figure 2:**
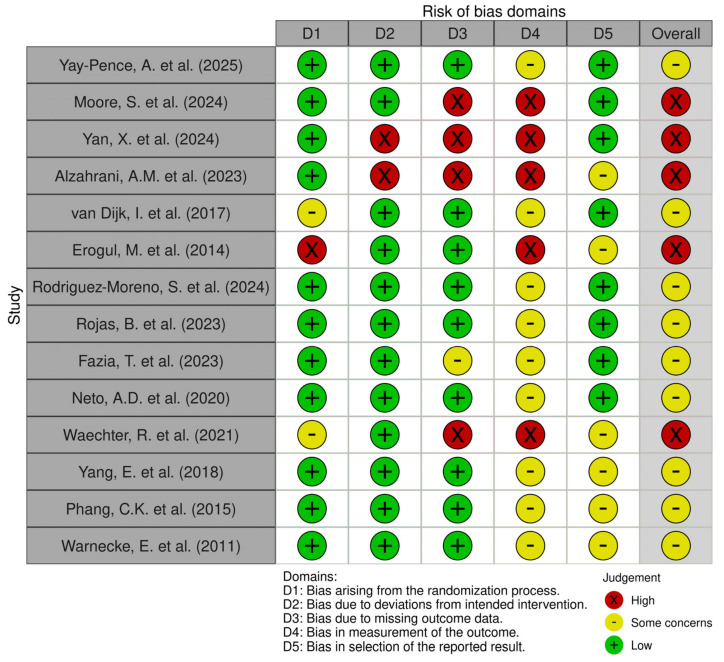
Risk of bias of the included RCTs using the Cochrane RoB 2 tool, represented in five domains with three levels of judgment (low risk, some concerns, and high risk) and the overall score for each study [6,7,8,9,11,12,15,16,17,20,21,23,25,26].

The graphical representation of the risk of bias of the RCTs included in this review is illustrated in Figure 2. Regarding the overall score, nine studies revealed some concerns, and five studies were classified as having a high risk of bias.

**Figure 3:**
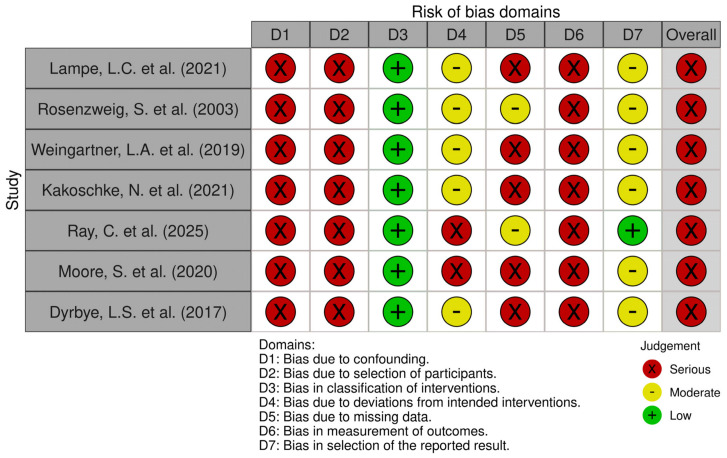
Risk of bias of the included non-randomized studies using the ROBINS-I tool, represented in seven domains with three levels of judgment (low, moderate, and serious) and the overall score for each study [10,13,14,18,19,22,24].

The graphical representation of the risk of bias of the seven non-randomized studies is illustrated in Figure 3. Regarding the overall score, all of the studies were classified as having a risk of bias.

## 4. Discussion

This systematic review evaluated the literature about the use and effectiveness of MBIs in reducing the psychological stress of medical students and improving other mental health outcomes, such as anxiety, depression symptoms and mindfulness skills. Notably, 13 of the 21 reviewed studies were published from 2020 onward, providing a more up-to-date analysis than previous systematic reviews. This allows the identification of emerging trends, such as mobile-based interventions and condensed programs, that are shorter than the traditional eight-week format. As to the former, mobile mindfulness meditation interventions, using mobile devices such as smartphones and apps, are increasingly popular and have shown effectiveness in reducing stress and anxiety and in increasing the well-being of university students [53].

The included studies evaluated multiple interventions such as the MBSR, the CCT, and other mindfulness-based programs. Overall, most studies reported reductions in perceived stress after the intervention, although the results were not entirely consistent across all studies. In addition to stress reduction, several studies also showed improvements in stress-related outcomes, including anxiety, depression, emotion regulation, and mindfulness skills. Notably, several studies failed to demonstrate the superiority of mindfulness interventions over control conditions [37,38,42]. Collectively, these mixed findings indicate that the precise mechanisms driving the putative effects of MBIs in medical education require further investigation.

MBSR was the most frequently evaluated intervention and most studies [32,34,37,38,39] reported reductions in perceived stress among medical students. These findings are consistent with previous systematic reviews reporting that MBSR may reduce stress levels and improve psychological well-being [54,55]. The current systematic review highlights a growing interest in cultivating compassion among medical students. The compassion-based interventions [18,40,41] decreased stress and improved mindfulness skills and emotion regulation. These findings carry significant implications for medical education, aligning with a growing body of research that demonstrates how compassion-based interventions enhance mindfulness and stress management among medical trainees and residents [56]. Recent evidence indicates that higher levels of self-compassion are associated with lower compassion fatigue and better mental health among physicians [57]. Herein, the effects of interventions promoting self-compassion should be tested in medical training contexts, both in medical students and trainees, with the aim of promoting well-being in future healthcare professionals.

Several studies included interventions that were not organized in an 8-week format. Some MBIs included adapted or shorter mindfulness programs delivered in non-presential formats. Since these interventions varied considerably in structure and duration, it is challenging in the present systematic review and with the results gathered to demonstrate the effects on perceived stress and related psychological outcomes among medical students. Studies with shorter durations (e.g., [46,50]) demonstrated some effects in reducing psychological stress and medical students have some time constraints. Herein, it will be important to focus future studies on the effects of shorter interventions. Prior studies indicated that online mindfulness programs can significantly reduce stress and improve related psychological outcomes [3,25,58,59]. The variations in duration, delivery format, and content likely influenced the outcomes. While shorter or online interventions provided practical solutions for students facing severe time constraints, it remains difficult to determine whether their benefits are sustained over time compared to traditional protocols. Regarding content, all types of MBIs, including MBSR, compassion-based programs, and brief formats, demonstrated reductions in perceived stress, although compassion-based programs yielded more consistent effects in stress reduction. All CTT programs showed a decrease in stress levels of medical students [18,40,41]. This is a finding that deserves to be further studied since compassion is important in medical care because it serves as a primary defense against clinician burnout [17,18]. The heterogeneity among the interventions complicates both a direct comparison of their effectiveness with structured protocols and the definitive establishment of a causal relationship.

Additionally, to implement MBIs in medical education contexts, it may be important to improve the experimental design of the studies. The evaluation should ideally follow a longitudinal approach, introducing foundational mindfulness skills during the preclinical years and providing reinforced sessions during high-stress clinical rotations. Furthermore, to overcome student resistance and perceived time constraints, these interventions should be embedded into protected time rather than added as extracurricular burdens. Flexible delivery models, including hybrid and online formats, may further improve accessibility and participation among medical students with demanding schedules. Future studies should evaluate feasibility and acceptability of the MBIs in medical schools, since this may be a constraint as previously shown by our research group [50]. By cultivating an institutional culture that prioritizes self-care and emotional intelligence, it is possible that medical schools can integrate mindfulness as a proactive component of professional identity formation, rather than merely reactive stress management [60,61,62].

Beyond the putative personal benefits, exposure to MBIs during medical training may have a profound ripple effect on future clinical practice. As students experience the tangible impact of these practices on their own mental health and resilience, they are likely to develop a deeper clinical intuition regarding the mind-body connection. This firsthand validation fosters greater sensitivity and openness toward integrative therapies, potentially increasing the likelihood that these future physicians will recommend or incorporate evidence-based mindfulness strategies into the care plans of their patients [60,61,62].

This systematic review has some limitations. The included studies were heterogeneous in what concerns the types and structure of the MBIs, duration, and delivery methods. Many studies had small sample sizes, which may reduce the statistical power and precision of the results, and the follow-up periods were short, making it difficult to determine whether the beneficial effects of mindfulness interventions were sustained over time. In addition, the included studies relied on self-reported questionnaires to assess psychological stress and related outcomes, which may introduce response bias, as blinding of participants was generally not possible. Several studies were also classified as having high or serious risk of bias, particularly non-randomized studies, due to methodological limitations such as lack of allocation concealment. High attrition rates and limited adherence to the intervention were also reported in some studies, which may have influenced the results. Furthermore, there was considerable methodological heterogeneity among the included studies. Most studies were single-center and had predominantly female samples, which limits the generalizability of the findings to all medical students and to medical schools with different programs or cultural contexts. Multiple scales were also used to measure psychological stress and secondary outcomes, making direct comparisons between studies challenging. Due to this heterogeneity, a meta-analysis was not conducted.

Overall, the findings of this systematic review suggest that MBIs could represent a viable option for inclusion in medical school curricula, as they may play a role in supporting student psychological well-being and mitigating perceived stress. These interventions could better equip medical students to navigate academic pressure and future clinical demands while fostering mindfulness and self-regulation skills. Since medical students usually face heavy workloads and time constraints, flexible online or hybrid delivery models may offer a practical means of supporting mental health. Nevertheless, we need to discuss the risk of bias of the included studies which may temper the confidence we can place in our overall conclusions. Chief among these is the high risk of performance and detection bias across 57% of the analyzed studies, mainly driven by confounding factors and selection of participants combined with a heavy reliance on student-reported outcomes. These constraints are common in studies using MBIs, as referred above [19,22]. In this type of interventions, the participants may become aware of their treatment allocation and potentially overestimate the intervention’s efficacy. Furthermore, the absence of active control groups in several of the analyzed studies mean we cannot rule out confounding variables or determine if the intervention outperforms current standard care. Consequently, while the initial trends are promising, our conclusions must be interpreted as tentative until validated by future studies with more homogeneous interventions, actively controlled trials. Based on the importance of promoting environments with better welfare in medical schools, it is important to continue the research of the effects of MBIs.

The present study outlines several gaps in the current research. For example, there is a lack of studies with long-term follow-up, the need for more objective outcome measures (like physiological stress markers), and the fact that most studies had mostly female participants. Future research should focus on larger, multicenter, and more diverse samples, use active control groups, and include longer term follow-up to assess the sustainability of the effects of MBIs on stress and stress-related outcomes. To measure stress levels, objective measures should be used in the future such as cortisol levels or heart rate variability to reduce the response bias associated with self-reported scales and strengthen the evidence. It would also be interesting to conduct studies comparing different types of interventions to determine which approaches are most effective, instead of more general MBIs. For example, future randomized studies should directly compare the different interventions, namely MBSR, compassion-based interventions, and brief or online mindfulness programs. The importance of using active control groups and standardized outcome measures, to clarify which formats provide the greatest benefit for medical students should also be considered in future studies. Moreover, future studies should evaluate feasibility and acceptability, not just efficacy. Strategies to improve adherence and reduce attrition should also be explored, and future studies should include more diverse populations, both in terms of gender and cultural background, to make the findings more generalizable.

## 5. Conclusions

This systematic review identified some studies showing the positive effects of MBIs in reducing perceived stress and improving related psychological outcomes, such as anxiety, depression, mindfulness, and emotion regulation, among medical students. Nevertheless, more research is necessary since this systematic review shows variable MBIs, methodological heterogeneity, small sample sizes, attrition, and reliance on self-reported questionnaires, which comes in line with our previous alert in the population of medical students [63]. Overall, MBIs may represent promising approaches to promote psychological well-being in future medical doctors and help prepare them for the demands of medical practice.

## Data Availability

No new data were created or analyzed in this study. Data sharing is not applicable to this article.
